# An HIV Narrative of Female Inmates With a Lifetime History of Mental Illness in Durban, South Africa

**DOI:** 10.3389/fpsyt.2021.637387

**Published:** 2021-08-25

**Authors:** Samantha Naidoo, Liezel Ferreira, Ugasvaree Subramaney, Saeeda Paruk

**Affiliations:** ^1^Department of Psychiatry, Faculty of Health Sciences, University of the Witwatersrand, Johannesburg, South Africa; ^2^Discipline of Psychiatry, School of Clinical Medicine, College of Health Sciences, University of KwaZulu-Natal, Durban, South Africa

**Keywords:** HIV, lived experience, South Africa, lifetime mental illness, female inmates/prisoners/offenders

## Abstract

**Introduction:** South Africa (SA) has one of the highest prevalence rates of Human Immuno-deficiency Virus (HIV) globally, with women carrying a larger burden of the disease. Furthermore, female inmates have higher rates of HIV compared to their male counterparts, with an over-representation of mental illnesses among female inmates as well. Additionally, mental illnesses are highly prevalent in people living with HIV, with HIV and mental illness sharing a complex bidirectional relationship. This study, which forms part of a larger two-phased, mixed-methods study, describes the experiences of contracting and living with/being affected by HIV, among female inmates with a lifetime history of mental illness, in a South African setting.

**Method:** This study was conducted at a correctional centre in Durban, KwaZulu-Natal, SA. Fourteen adult (18 years and older) female inmates, were purposively selected to participate in individual, in-depth semi-structured interviews. Participants had a lifetime history of mental illness, trauma and were either living with HIV, or affected by HIV. Women from diverse cultural backgrounds, who were fluent in English, were selected. This manuscript focuses on the description of the HIV component of the qualitative interviews only. Thematic analysis was used to analyse the data.

**Results:** Themes related to contracting HIV included intimate partner betrayal, gender differences regarding sexual behaviour, fear associated with HIV and the importance of pre- and post-test HIV counselling. Themes related to living with/being affected by HIV included the challenges women experienced in their home community, which contrasted with their experience of living with HIV in the prison community, and the importance of accepting an HIV positive life.

**Conclusion:** HIV is prevalent in the female inmate population at this correctional centre in SA. This study emphasises that whilst incarcerated, attempts should be made to educate, train, support and manage HIV in this population, thereby helping to curb the epidemic. Further research should aim at exploring such strategies. The study also underscores the importance of the continued need for HIV education in order to eradicate associated stigma and discrimination which are still prevalent in SA.

## Introduction

Although Human Immuno-deficiency Virus (HIV) is a global public health challenge, the majority of people living with HIV/AIDS (PLWHA) reside in sub-Saharan Africa (1). South Africa (SA) has one of the highest prevalence rates of HIV in the world with only Eswatini, Botswana and Lesotho exceeding HIV prevalence rates in SA (2). Approximately 7.7 million South Africans were PLWHA and the country had an HIV prevalence of 20.4% among adults aged 15 to 49 years in 2018 (3). SA recorded 240,000 new infections in 2018 and 71000 people died of AIDS related illnesses in the same year (3). Although the incidence of HIV is declining, the prevalence is increasing, since more PLWHA are on anti-retroviral therapy (ART) and are living longer (4).

In sub-Saharan Africa women have a higher prevalence of HIV than men (5), and in SA, women are disproportionately affected by HIV with 62.7% of PLWHA being women (3). Cultural practices, gender-based violence and the low socio-economic status of women are all cited as reasons to explain this disparity (5). The HIV prevalence among young women aged 15 to 24 years is almost double that of young men of the same age (3.83% in males vs. 7.25% in females) (6). The epicentre of South Africa's HIV epidemic is KwaZulu-Natal (KZN) province (7). Although KZN is the second most populous province in the country (Gauteng being the most populous) it carries the largest burden of HIV (27%) (7). However, the burden of HIV and mental illness among female inmates in KZN correctional centres remains under-researched.

While women constitute a minority of the total prison population, the number of incarcerated women has increased by 50% globally since the year 2000 (8). Furthermore, in most countries the prevalence of HIV in prisons exceeds the prevalence of HIV in the general population (9). A recent review of HIV prevalence in prisoners found that, in some countries, female prisoners had a higher prevalence of HIV than their male counterparts and this is particularly concerning in West and Central Africa, where the prevalence of HIV in female prisoners is almost double that of male prisoners (9). Women are at higher risk of entering prison with sexually transmitted diseases and HIV/AIDS (10). Previous estimates of HIV prevalence in South African correctional centres were as high as 40 to 45% which is more than double the prevalence amongst adults aged 15–49 years in the general population (11). In addition, women in prison have elevated rates of mental illness compared to their male counterparts (12). Mental illnesses are highly prevalent in PLWHA (13), with HIV and mental illness sharing a complex bidirectional relationship (14, 15). Mental illnesses may increase an individual's risk for contracting HIV due to increased social vulnerability; increased inclination for high risk behaviour; associated alcohol and substance misuse; and disinhibition within intimate relationships. Conversely, mental illnesses may be secondary to direct result of HIV neuro-invasion or psychosocial ramifications of living with a chronic illness, or due to adverse effects of antiretroviral therapy (ART) (14, 15). Despite this high prevalence of women living with HIV (WLWH) in SA and the elevated prevalence of WLWH in prison, there remains a paucity of qualitative studies that provide an in-depth understanding of their lived experiences, particularly among inmates with mental illness.

A further area of interest of this study was incarcerated women's HIV experience within the South African cultural context. SA is a country made up of people of diverse cultures and ethnicities. The United Nations Educational, Scientific and Cultural Organization (UNESCO) defines culture as a set of distinctive spiritual, material, intellectual and emotional features of society or a social group, that encompasses, not only art and literature, but lifestyles, ways of living together, value systems, traditions and beliefs (16). Ethnicity refers to shared cultural practices, perspectives and distinctions that set apart one group of people from another. The most common characteristics distinguishing various ethnic groups are ancestry, territorial possession, language, forms of dress, a sense of history and religion (17).

The largest ethnic group in SA is the Zulu nation, the majority of whom live in KZN and Gauteng provinces (17). A study in four South African correctional centres found that cultural conceptualizations influence health-seeking behaviour among inmates, and that both consultation of traditional healers and biomedical remedies is widely practiced (18). Traditional beliefs, medicine and health practitioners play an important role in healing in the lives of African people (19). It is therefore important to examine female inmate's lived experiences of HIV within this African cultural setting.

The first phase of this study, which measured the prevalence of mental illness, HIV and trauma in 126 female inmates at this correctional centre in KZN, found that 36.5% of women had experienced a psychiatric disorder, or a relapse of their psychiatric disorder in the past year, while 90.4% of the women had a lifetime history of suffering from a psychiatric disorder (20). In addition, in phase one of this study, 64.3 % of the participants interviewed were WLWH (20). Phase one also revealed an association between HIV and post-traumatic stress disorder and between HIV and alcohol use disorder (20).

The aim of this manuscript was to explore the HIV narratives amongst female inmates with a lifetime history of mental illness in order to gain an in-depth understanding of their lived experiences and perceptions of HIV, in a South African setting.

## Materials and Methods

The findings reported in this manuscript form part of the second phase of a larger mixed methodology, sequential, explanatory design study which aimed to describe the mental health needs of female inmates in Durban, SA. This study, in keeping with a design as described by Creswell and Plano-Clark (21), began with a quantitative analysis. A qualitative arm, which was used to explain findings from the quantitative phase (i.e., the high prevalence of trauma, HIV and mental illness), followed and helped to contextualize these quantitative findings. It enriched the quantitative findings and generated new data. The qualitative phase was conducted in keeping with a constructivist epistemology.

This study also adopted a transformative framework (22). Transformative research helps to create a more just and democratic society. The transformative lens can be applied to taking a stand on a broad array of issues. In this study, the research is done through a social justice and feminist lens. The results of the research are intended to contribute to broader social objectives which include serving as an evidence base to create an awareness of the mental health needs of this marginalised population, and to inform future policy development for rehabilitative programmes for female inmates in the Department of Correctional Services (DCS) in South Africa.

This study was conducted at a correctional centre in KZN in SA, which is one of the largest correctional centres in sub-Saharan Africa. It accommodates mainly male prisoners but it also has a section for females. Females are referred from many parts of KZN as this is one of the only correctional centres in the province which accommodates women serving life sentences.

### Participants

In phase one, 126 female inmates were recruited into the study and their prevalence of mental illnesses, trauma and HIV were quantitatively measured. After analysis of phase one data, 14 women (including sentenced offenders and remand detainees) were purposively selected and invited to participate in phase two, which took the form of individual, in-depth semi-structured qualitative interviews. The 14 women, who were from diverse cultural backgrounds, had a lifetime history of mental illness and trauma, and were either living with, or affected by HIV. This manuscript reports on the HIV component of the qualitative interviews. Including women who were not living with HIV, broadened our understanding of perceptions of all incarcerated women regarding HIV. Only women who were fluent in English were selected to participate in phase two as the qualitative interviews were conducted by the first author in English. When no new themes emerged from the data with respect to HIV narratives, saturation was achieved and sampling ceased.

### Data Collection

The first author, who conducted the data collection and analysis, is a forensic psychiatrist and completed training in qualitative research at the University of the Witwatersrand. The interview followed an open-ended format with a few questions on each topic (HIV, trauma and mental illness) in the interview guide and with minimal probes. Participants were encouraged to speak freely and openly about their lived experiences. The semi-structured interviews were audio-recorded.

### Ethical Considerations

Ethical approval was obtained from the University of the Witwatersrand Human Research Ethics Committee (Clearance number M181026) and approval was also granted by the DCS in SA. The study was fully explained to the selected participants and written informed consent, including consent for audio-recording, was obtained.

### Analysis

After verbatim transcription of audio-recordings, the transcripts were read more than once to verify accuracy with the audio-recordings. Braun and Clarke's thematic analysis was the chosen method of analysis (23). Data analysis commenced with the first author familiarising herself with the data by reading through all the transcripts several times before commencing the coding process. A qualitative data analysis software programme, MAXQDA, was used to analyse (code) the data (24), which also ensured an electronic audit trail. A code book was then developed using MAXQDA. After the 14 transcripts were coded, the researcher began the process of compiling initial subthemes and later themes. Themes were revised multiple times and were subsequently correlated with actual extracts of quotations from participants which highlighted the theme. This was an iterative and cyclical process and was done in collaboration with the second author, a qualitative researcher and clinical psychologist. Idiomatic expressions were retained in the quotations. Participants are identified by pseudonyms in the manuscript.

Credibility, dependability, transferability and confirmability (which includes reflexivity) as defined by Guba and Lincoln were the constructs used to establish scientific rigour (25). Credibility was ensured by the first author conducting all the interviews, as well as through analyst triangulation by the first and second authors. Transparency was enhanced using a qualitative data analysis software programme. Thick descriptions of the study setting and population as well as detailed in-context descriptions facilitated transferability of the findings.

## Results

### Socio-Demographic, Clinical, and Forensic Profile

The mean age of the 14 participants was 36.2 years (standard deviation 9.3). The majority of women had a high school level of education however, a large proportion were unemployed prior to incarceration. Most participants were from urban areas and were single, separated, divorced or widowed. The majority of women were living with HIV and were on anti-retroviral therapy (ART). Women were charged with offences including fraud, theft, possession of drugs, murder, robbery with aggravating circumstances and kidnapping. The most common lifetime mental illnesses among participants were major depressive disorder, post-traumatic stress disorder and alcohol use disorder. There was also an over-representation of borderline personality disorder in this qualitative phase sample.

### Themes

Significantly, 27 years after the new democratic dispensation in SA, remnants of the destructive effects of apartheid (national policy of racial segregation) still linger (26). This was manifest in the strong racial sentiments which permeated the women's narratives. The relevant quotations were thus included in the manuscript to highlight the racial stereotypes and prejudices still present in SA today however, the actual words describing specific ethnic/cultural groups have been redacted. The authors dissociate themselves from any prejudicial sentiments reflected in statements harboured by the participants.

The themes are summarised in Table 1 and then described in detail.

**Table 1:** Summary of the main themes and subthemes.

**Overarching theme**	**Theme**	**Sub-theme**
Contracting HIV	Intimate partner betrayal	Disbelief and anger
		Injustice of being infected
		Frustration at partner's denial and lack of support
	Gender differences in sexual behaviour	Men having multiple sexual partners accepted
		Women are raised with traditional conservative values
	Fear associated with HIV	Fear associated with potential loss of physical integrity or loss of life
		Fear of contracting HIV in prison
	Importance of pre- and post-test counselling	
Living with HIV	HIV in the home community	Reluctance to disclose their status
		Lack of knowledge, misconceptions and prejudices about HIV
		Stigma and discrimination
		Cultural beliefs about HIV causation
	HIV in the prison community	Disclosure
		Support
	Coming to terms with an HIV positive life	Acceptance of diagnosis and starting treatment
		Rationalising their illness
		Adopting a healthy lifestyle

## Contracting HIV

### Intimate Partner Betrayal

#### Disbelief and Anger

Many women described being shocked at their diagnosis, as they expected their intimate partners to be faithful to them. Sibongile expressed her disbelief upon diagnosis as she had never engaged in sexual intercourse prior to marriage, “My husband…he was my first one, I didn't sleep with my boyfriend in high school…and then now I'm married, pregnant and then I'm HIV positive…I was very angry. I was shocked.” Esther was enraged when she discovered she was HIV positive, “I think if there were not any walls here or any burglars or any cage holding me back, I was furious…I was very, very angry.”

#### Injustice of Being Infected With HIV

Didi expressed her desire for vengeance at being betrayed by her husband and the injustice of being infected since she had been a faithful wife, “I know I was a good woman, I never cheat but now I find that I am HIV positive.it was so difficult. I could not even like my husband, I was feeling even to fight back because he was the one…I was so trustful, I was so faithful to him.” Katlego also spoke about her feelings of being unjustly infected by her partner, “I never pursued the disease, in a sense as whereby I had a lot of sexual partners, but instead I got it from the one person that I trusted.” Noma described how many women felt about being infected by their partners, “Most of them, especially the married ones, they feel that they have been robbed by their husbands because most of…women, they don't go around cheating. So, most of our…men, going around and cheating, it's kind of a norm. So, you'll be sitting at home doing everything by the book, then your husband will be going around, then he will bring the HIV to you. So, that is what is happening…because most married people are HIV positive and the carrier is their husbands.”

#### Frustration at Partner's Denial and Lack of Support

Some participants explained that they had to cope with denial from their intimate partners and in some cases the women had to force their partners to get tested. Some assumed the role of a care-giver for their partners, whilst simultaneously having to come to terms with the illness themselves. This was manifest in Noma's account, “The minute you tell him that you're HIV positive, their attitude, they just snap and say ‘No, you're the one who brought this', and they don't want to go and test…so, you have to gradually beg him to go and test so that you can start treatment together. So, i-role iyakho [your role] it becomes more of a nurse, more of a counsellor whilst you are also trying to figure out what to do about this. Whilst you're trying to adjust that now, I'm HIV positive whilst I'm being sincere and honest to my husband. The very same husband that brought this to you is also your responsibility.” Sibusisiwe also expressed her frustration and disappointment at her partner's denial, “He was the cause of it and he was busy denying it and at times I would think what kind of a person he is who doesn't own up or doesn't man up and you know [say], ‘I've done it, I'm sorry' you know, whatever, you know, he just denies everything.”

### Gender Differences in Sexual Behaviour

#### Men Having Multiple Sexual Partners Accepted

Some women alluded to infidelity among men being accepted as a norm and that this was frequently how HIV was introduced into the relationship. Noma expressed the following, “It [cheating] is a norm, Doc. If you're not doing it, it's as if you're not an ordinary man…If you're married, you must be married, then you must have a concubine on the other side. If you're not doing that, you're not man enough. That is a norm…if you're having a problem with your husband, they will tell you that, ‘Even my grandfather, even my great-grandfathers, that's how things were done in an…way'. It's a norm.” Didi stated that her husband's family practised polygamy and he wanted to practise the same in their marriage, “Because he grew up in a family of a polygamy so he wanted to apply that how he grew up to me.” Katlego described her father as having other partners while being married to her mother, “My mom and my dad were married, yes, but he was in and out of the picture. He had other women.”

#### Women Are Raised With Traditional Conservative Values

Some participants stated that women were raised with strong, conservative, traditional values. They were raised to believe that they had to remain celibate until marriage, as Sibongile commented, “Yeah my culture plays a very important part…because I was told while I was still very, very young ukuthi [that] that you don't sleep with a man…you can have a boyfriend, but there's no sleeping with the boyfriend up until you get married, up until you have somebody whom we know ukuthi [that] okay this is your man; he must pay for your family and then you can have your home, you can start to have your children and everything. That is how I grew up.” Similarly, Katlego noted, “We're taught that from a young age that you keep your virginity for as long as you can. That's your present to your husband, when your family gives you away to the next family.”

With regards to the traditional role of women, participants described gender inequalities that persist in their culture as evident in Noma's comment, “In most African countries or cultures, the woman must always be submissive. That is the problem…They were conditioned to think that way and to act like that.” Didi also described the traditional view of how a woman should be raised in her culture, “The role of the woman in that [deep rural] area, from the age of sixteen, eighteen when you have got an ID [identity document] you must get married, that is what they know. And when you are able to write a letter then it is how your schooling must stop. There is nothing more that a female must be more educated. You must have children, must have your family.” These accounts, as described by the women, portray themselves as being disempowered and subject to a patriarchal society.

### Fear Associated With HIV

#### Fear Associated With Potential Loss of Physical Integrity or Loss of Life

Women spoke about their overwhelming fear, upon diagnosis of having HIV, related to becoming severely ill and thoughts of imminent death, fuelled by memories of people from their past who had suffered such a fate. Didi feared her physical deterioration. She said, “When I look at my neighbours how they are facing this vulnerable disease, because some, they could not eat. Some they were full of sores. Some the legs were swollen. They have got a problem of rash. So, I was looking at the diseases that I will be facing, a giant. That is why I was so having that fear that ‘Oh, it's me, it's my turn.”' Katlego also expressed fears of premature death due to HIV, “Because I always used to see it as this disease whereby you'll get sick and drop dead…it's scary because to me, I thought it was a life sentence. I was just going to die at any time.” Melissa related the same feeling of being overwhelmed with fear upon diagnosis, “It was terrifying. For me at first I thought it was just over you know.” The fear of impending death was also evident in Nokukhanya's account, “When I went to the clinic here, they counselled me…I even told the person that was counselling me first that I'm too scared to get the result because I used to see my aunty getting sick, so I will be like that and I will die in prison.”

#### Fear of Contracting HIV in Prison

A few of the women who were HIV negative stated that prior to coming to prison they had never interacted closely with family or friends living with HIV. They spoke about having many fears about contracting the virus in prison which was mostly due to a lack of knowledge as described here by Neeta, “Outside I didn't come into contact with people who are HIV positive, whereas here, I have. When I first came to prison, I was very cautious, you know, because [of] us using the showers together and all of that, it terrified me…so yeah, I was scared.” Alicia, shared similar sentiments, “I have not come across someone close to me within my family or within my friends that are HIV positive…I was terrified of course of HIV. I didn't want to use the same anything as them [inmates living with HIV] because I thought, ‘Oh my gosh, I could get it.”'

### Importance of HIV Pre- and Post-test Counselling Upon Diagnosis

Many participants spoke about the importance of HIV pre- and post-test counselling at the time that they were diagnosed. Women who did not get any counselling described how difficult the experience was and what a devastating impact the diagnosis had on them, because of their lack of knowledge about HIV as described by Katlego, “It was horrible. It was basically one of the worst moments of my life, whereby, what can I say, maybe being on the outside you get counselling, pre-test counselling, here there isn't most of that, so you're just thrown into the deep end of something that you don't know about and it's scary. Because to me, I thought it was a life sentence. I was just going to die at any time so maybe if I got pre-counselling and they told me that you know, this is the disease and this is what happens, you get treatment, then maybe I think I would have gotten, I would have taken it a little bit better.” On the other hand, women who did get HIV pre- and post-test counselling described how this helped them accept and cope with the diagnosis as detailed by Noma, “The counsellor, I think he did a lot. The way he counselled me, he prepared me. He prepared me pre- and post. The time I left there, I did not have any regrets about testing, even the results, I just accepted them…I think the person who was counselling me was good enough because he told me that it's not the end of the world.” Katlego also expressed the desire to become an HIV counsellor so she could help support others, “I like learning about things even if it was HIV courses whereby I could maybe learn to be an HIV counsellor and help someone else.”

## Living With HIV

### HIV in the Home Community

#### Reluctance to Disclose Their HIV Status

Many women discussed how difficult it had been for them to disclose their status to their families and friends while they were living in their home communities. Sibusisiwe expressed the need for PLWHA to conceal their status for fear of being judged and disparaged, even by those closest to them, “While I was outside, you have to be discreet, very discreet with what you do and how you do it, who sees you and who doesn't, because even in your own family, people will talk…and belittle you, and you feel like you, you [are] nothing.” Melissa echoed this, stating that her lack of disclosure was due to fear of rejection and possible loss of relationships, “I would like to be open about my status. Currently I'm not though. I think I am also afraid of people judging me. Yes. I'm afraid of the rejection…like people not wanting to be around me maybe if they know that I am HIV positive.” Didi also expressed that people's reluctance to disclose was driven by their fear of rejection by those they loved, “People, they are afraid to disclose their status because they know that they will lose something. They will lose their marriage. They will lose their friendship. They will lose even their children.” Due to the stigma attached to HIV, Sibongile, a teacher, spoke about the need to conceal her status with regards to taking ARTs, “Like maybe you are going for a [matric] marking and I had to pack my medication, you see, and you have to drink your medication. So many people are there, you have to use those pill containers…so that people they won't see you carrying those containers that are written ama [the] antiretroviral and everything, you have to put them into those pill containers like you are drinking pills like everybody else is drinking pills.” Mpumi concurred that people are hesitant to disclose their status because of the stigma attached to the diagnosis “Most people hide their statuses yeah, even getting into relationships, people hide their statuses. I've dated a few guys that have hidden their statuses from me, you know, and [you] end up finding ARVs in their cupboard, somewhere along the line during the relationship. Yeah, people still hide their statuses most of the time because it's still a stigma.” This proved to be the case among women from all cultural backgrounds, as seen with Lisa, “Like in the…community, they still hide the fact that they're HIV positive.” Didi alluded to people's reluctance to disclose their status impacting on their treatment adherence and ultimately resulting in negative effects on their health outcomes, “But outside it is hard to disclose…people have got their secrets, their confidentiality so that is why people they are not healed…because that is where [why] they do not take their medication on time.”

#### Lack of Knowledge, Misconceptions, and Prejudices About HIV

Participants related how the lack of knowledge about HIV was still very rife and that this led to misconceptions and prejudices. Participants described how they would be treated by the community if they revealed their HIV status. Didi stated, “The problem is that, if you disclose your status to the outside community…they cannot share their food with you. There will be no contact…there is that myth of HIV, using my spoon, using the same toilet then you will be transmitting the HIV virus…They cannot even hug, if you hug a person where [when] they know you are HIV positive they say ‘Oh'. That is the problem. It is hard because they lack knowledge.” Katlego also remarked that there was a lack of knowledge about HIV in her community and that people were reluctant to openly engage in discussions about HIV, “The…community from what I know is not really educated about HIV. They're blind to it. It's there but it's not something they like talking about. It's not something they're educated about because it's automatically like the mentality that I had, which is automatically when you're sick, you're gonna die when you're infected.” Lack of knowledge and misconceptions about HIV prevailed across women from all cultural backgrounds. Lisa commented, “I am from the…community, they're…very ignorant [about HIV].” Alicia admitted to a lack of knowledge as being responsible for her prejudices, “I would ostracise them [PLWHA] because I had no knowledge of it. Now I've got more knowledge of it and I see it differently.” Esther also shared similar thoughts about the misconceptions that existed among people in her community, “…people are very naive because they only believe I'm sure that it's just amongst…people, which is utter nonsense because at [in] this day and age anybody can get HIV.”

#### Stigma and Discrimination

Women described that they felt judged by society for being HIV positive. They quoted derogatory terms that were often used to describe WLWH and stated that the community would often blame the women for contracting the virus, as participant Sibusisiwe remarked, “They would think that maybe you [are] a whore of some sort. That you sleep around that's why you have HIV.” Mpumi who was HIV negative also felt the same way, “But there's still that stigma, you know, having HIV sometimes people think that…the women that are HIV positive probably sleep around and that's why they got it, they deserved it or something.” These sentiments were evident across cultures, as illustrated by Neeta, “The…community…they just assume you were sleeping around and that's how you became HIV positive…the moment you have this HIV positive status, you have a stigma attached to you that you're from the lower end of life and you're the trash of the world.” Similarly, Alicia commented that women received disrespectful labels and were blamed for being infected with the virus, “If it were to happen to someone who's maybe…, it would be frowned upon as maybe something dirty…I thought it was a dirty thing and I am assuming that's what everybody else in my family or in the culture would feel as well…it would be self-inflicted, that's what I thought.” This was also expressed by Seleste, “They [people from her community] think that you sleep around with…people and that you are a disgrace to the family. You are not part of the family because you slept around with so many guys and did so many wrong things and they cannot accept you in the family and all kind of things. And they always just give you bad names and everything.” Melissa remarked, “If they [people from her community] know that you're HIV positive, it's like a death sentence. Yes, so it's like you're the walking dead. You probably don't exist anymore…they would normally treat a person like that like trash, you know.”

Participants also quoted non-verbal examples of discrimination which they encountered regularly. This enacted stigma evoked feelings of loss of worth and dignity, as detailed by Didi, “When you are taking your ARVs in the centre…so people they look at you, they name you. You are just stigmatised, there is that discrimination, ‘This woman is taking the ARVs'…there will be spreading of news that our teacher is HIV positive…so this giant of being HIV positive, people they are still not accepting.” This was echoed by Sibongile, “Like the look nje [just], even by not telling a word, like…outside the doctor will give you a script for 6 months. So you just take that script, you give it to the pharmacy, maybe there are so many people waiting in the pharmacy, they will say, ‘Oh, ARVs, those are ARVs.' Just the look, only the look will tell you a lot without even speaking. So you will see that one is so judgemental.”

#### Cultural Beliefs About HIV Causation

Women talked about how HIV was perceived, particularly in the rural communities, as being a spiritual illness due to bewitchment rather than as a medical illness. They would thus seek intervention from traditional healers rather than western medical practitioners, as described by Didi, “In my [rural] community there are two groups. Others, they say you are HIV positive, there is nothing of a such. They say if a person is HIV positive, it is only someone that is using the muti [African traditional medicine] to make that person sick. If you have got a problem with the legs, swollen legs, there is something that has happened. Maybe it is this spiritual ancestors, if [so] they want to take you to a spiritual sangoma [African traditional healer]. So, there is that demonic spirit that has come into your life to change you.” Noma concurred with this notion, “Like, there are people who go to Joburg to work in the mines. So, when they come back, some of them they come back critical, sick, sick, sick [with HIV]. Then they [family] will take them to the sangomas and then they will say ‘No, that person has been bewitched,' unedhliso [poison], and all those things. Then, taking the person to the clinic will be the last resort, but maybe by the time they take the person to the clinic, it will be too late. So, they rather go to the traditional healers than to the medical professionals.”

### HIV in the Prison Community

#### Disclosure

The women spoke about it being easier to disclose their status inside prison for many reasons. Outside prison many women felt as though they were alone, as if they were the only ones infected with HIV, whereas in prison, they saw that many women were living with the virus and this encouraged them. This was expressed by Katlego, “There are so many of us living with it here. The majority of us here have it…I think with me being…diagnosed in prison, was in a way I think a blessing because maybe if I was on the outside, I would have still been in denial because I would have thought it's only me that has HIV, but being in prison, I saw that there are women living healthy lives, looking healthy and alive with HIV.” They stated that they did not feel judged like they did outside and that they felt more accepted. Sibusisiwe explains, “When you get here, it's unlike on the outside. On the outside world you feel everybody's on you, watching you and whatever you do, you must hide this, you must hide that. There's no judging here inside. When we go to the clinic, we go all to the clinic, we go to get our medication. No one says ‘Oh this one is taking ARV'. No one is on anyone's case. You just do you.” Esther shared a similar perception, “So, here it's no big deal because a lot of people here are [have] HIV, I can't say everybody because I don't know but you'll find that the way we know is when we fetch our medication which is obvious so that's the only way you're going to know.”

As alluded to above, due to the lack of privacy in prison, all WLWH attended the same clinic every month to consult with the doctor and collect their ARTs, thus the women's status were revealed due to the nature of the prison system. Most women felt that this unintentional disclosure had a positive effect as the women did not feel alone. It encouraged adherence, they were able to enlist the support of other WLWH and it helped to eradicate stigma, as described here by Noma, “Then, you cannot hide it in prison. Like, what is happening, we are grouped okay, every Tuesday and every Friday there is a chronic clinic. They will call your names, ‘So and so, and so, you're seeing Doctor H'. Everybody will know that Doctor H is the doctor for HIV, so you cannot hide it. Even if you hide it, you're staying with a roommate. At six o'clock, she will see when you're taking tablets and the container for HIV is very loud, you cannot hide them so, you cannot hide it…I think it's good because the more people know about you and now you don't mind, I think it's taking that stigma away. How I wish it can be like that even outside. Here, it's different from outside. There is more acceptance of HIV than outside.” She elaborated on the positive side of being in prison which was that it encouraged good adherence which translated into better health outcomes for inmates, “I think it's better here than outside and I've noticed that most people who are in prison are taking ARVs better than people who are outside.”

#### Support

Contrary to the participant's experience in the home community, most of them found the prison community to be very supportive. The women spoke about the importance of supporting each other with respect to their HIV status, as a coping mechanism for living with the illness. Didi detailed this, “If you are using the medication, we support each other. Even if the person does not have the medication, we even supply for each other if we are using the same medication. We remind us [each other], ‘Time, tablets time'. So, we even bang the walls, ‘Six o' clock'…It is good here in prison to disclose the status because we are altogether in one section, in one building for many years…so we must support each other because some other people, if they are having some problem, they do not take their medication, they do not take their food. So, we help each other to make us strong.” The women alluded to support not only from other inmates but also from the staff who encouraged them, as Nokukhanya remarked, “Even if you lose weight, they [staff] will be more concerned, ‘Have you seen that you losing weight? Did you take your CD4 count? Did you take your medication regularly?”'

Participants also identified gaps in the current correctional system with respect to comprehensive management of their HIV illness. They expressed the need to establish support groups both inside and outside prison as described by Katlego, “I think I will gather information once I'm out of prison because here there aren't any of those facilities. There are no support groups, like no one to talk to about it basically. Yes, there are many of us that are positive, but it's every man for themselves basically. You just go to the hospital, you take your treatment and goodbye, that's it…maybe having someone that can tell me, ‘Listen, I've had it for this long. I'm alive and kicking. I'm fine' would bring me more comfort. So, support groups to me, would mean a lot.” Sibusisiwe went on to explain further how support groups would encourage and assist women to start treatment, eradicate their fear of ARTs, encourage adherence and how it would help women by instilling hope that they could live long and healthy lives, “There are people also who are here, maybe they don't want to take their medication and they don't understand and if I come to you, you see I look healthy and strong, and the person who doesn't know me, that I'm taking medication wouldn't even tell that I'm HIV positive…I would encourage that person to take the ARVs, tell them that it's going to be okay, it's their life and it's important for them to do as they are still going to live longer and they can see that you also look healthy…and strong and then they get motivated.”

### Coming to Terms With an HIV Positive Life

#### Acceptance of Diagnosis and Starting Treatment

Many women described taking a while to accept their HIV positive status but, after acceptance, they decided to start ARTs as stated by Katlego, “It took me a while to come to terms with it but eventually I thought I'm infected, I'm infected, I might as well take treatment and try and deal with it.” Sibusisiwe shared similar sentiments, “Now I feel I've accepted and I'm on ARVs and I'm healthy, I'm strong…I adhere to the times how I'm supposed to take the medication, so I've dealt with it.” Noma summed it up by saying, “So, taking treatment is the best thing that you can do for yourself.”

#### Rationalising Their Illness

Many women rationalised their HIV by comparing it to other chronic illnesses and some even stated that they preferred having HIV, as it had a better prognosis compared to other illnesses such as diabetes or cancer as described by Noma, “The only difference between myself and the other person who is not HIV positive is that I have to take medication. And HIV, the way I look at it, it's better than cancer, because the minute you start taking ARVs, your viral load goes down and you can still live a normal life. Unlike cancer which is a silent killer. So, I think HIV, I think I like my HIV.” Esther shared similar thoughts as well as the fact that HIV had a very simple treatment regimen which facilitated good adherence, “No, I mean my mum…was diabetic so there was a possibility of either having diabetes…which is a worse killer than HIV…There is only one tablet that I take daily so it doesn't affect me at all…I think diabetes is worse than having HIV really…. So, when you say you are HIV positive it's like okay. It's nothing really.” They spoke about how HIV was not a huge problem in their lives, because they had other more stressful issues to deal with being incarcerated, as expressed by Sibongile, “HIV is just a small thing compared to the things that we are facing every day. There are so many things and so many problems around here, so…the HIV thing it is not a problem.”

#### Adopting a Healthy Lifestyle

Women also spoke about adopting a healthy lifestyle and making positive changes in their lives after accepting the diagnosis, as mentioned by Noma, “But now that I discovered that I'm HIV positive, I started changing my lifestyle, changing the diet, starting taking care more of myself and choosing a better diet, multivitamins and stuff.” Nokukhanya also started making better choices, particularly concerning alcohol use and safe sex practices, “I can say it's changed the way I used to live. I never used to care for myself. I used to drink [alcohol] outside, I used to sleep with women not knowing their status…I don't do that without knowing your status and before I do that, I tells you my status, so let's have condoms.” Seleste shared a similar experience with respect to her illicit drug addiction, “It [HIV] did change my life…It makes me think of not doing the things that I did in my past, like going back to drugs.”

## Discussion

This article situates one of the quantitative findings from the first phase of the study, that is, the high prevalence of HIV among female inmates, in a South African setting, and it explores the impact of this illness on the lives of these women, both before and during incarceration. In the narratives of the participants, themes pertaining to contracting HIV and also living with HIV were elicited. These themes revolved around intimate partner betrayal, gender differences regarding sexual conduct, fears surrounding contracting HIV and the consequences thereof, the importance of HIV pre- and post-test counselling, the experience of living with HIV in the home and in the prison community as well as coming to terms with an HIV positive life.

A strong theme of intimate partner betrayal was apparent, with many participants describing that they were unknowingly infected with HIV by their husbands or boyfriends. They lamented the injustice of being infected in this manner as they had trusted their intimate partners. Women spoke about their emotional experiences upon discovering that they were HIV positive, which included feelings of disbelief and anger. To add to their distress, they described their partner's denial of accountability and their frustration at having to force their partners to test for the virus. Similar negative reactions were cited by Maman and colleagues in a South African study in which male partners were reported to have overtly negative reactions when their female partners disclosed their own positive status (27).

Participants also alluded to their cultural background with respect to the common practice and social acceptability of men having multiple sexual partners, whether as part of polygamous relationships, or secondary to infidelity with married man having concubines. They also described the generational pattern of this practice. This is supported by the literature. Shisana discussed this practice whereby in some cultures in southern Africa, men are expected to have multiple partners, while women are expected to be monogamous (5). This finding which related to men more commonly having multiple sexual partners was further reiterated in a South African study by Onoya et al. (28). Multiple sexual partners also represented the main cultural practice cited by participants as being a reason for the spread of HIV in a qualitative study conducted in Lesotho (29). This community-based study from Lesotho detailed this phenomenon which makes reference to the African culture accepting men having multiple sexual partners, but expecting women to have only one partner (29).

The women in our study spoke about the values which were inculcated in women such as the traditional gender roles and expectations of women which manifested in women feeling disempowered in relationships. Some African communities remain patriarchal, which contributes to gender inequalities in relationships, and by extension, the sexual relationships between men and women (5). This leads to women feeling unable to express and assert themselves with respect to issues like safe sex practices, which makes them more vulnerable to contracting HIV and has been cited as one of the reasons women in sub-Saharan Africa are disproportionately affected by HIV. Gender inequality thus drives the HIV pandemic (30).

Fear associated with HIV was also a common theme expressed in this study. WLWH reported experiencing an overwhelming fear associated with being diagnosed with HIV which was related to the potential loss of physical health and imminent loss of life. Women who were HIV negative spoke about their fear of contracting HIV in prison which was largely due to a lack of knowledge about HIV transmission. A recent qualitative community study in the North West province of SA also described prominent fear, among community members, of contracting HIV from HIV positive individuals (31). This fear, as was the case with our study participants, was rooted in their lack of knowledge regarding transmission. This underscores the continued need for education of the general population and incarcerated populations about the transmission of HIV.

WLWH in our study also highlighted the importance of HIV pre- and post-test counselling upon diagnosis. They detailed the devastating impact of the absence of counselling on acceptance of, and coping with the illness. HIV counselling and testing remains the gateway to all strategies related to the care, treatment and prevention of HIV infection (32, 33). Counselling and testing is crucial in not only helping those who test positive to come to terms with their illness, but it is also critical in bringing the rampant scourge of HIV under control, particularly in sub-Saharan Africa. In addition, some women also wanted to learn more about the illness and expressed a desire to become HIV counsellors so that they could educate and support other women both inside and outside prison. Empowering female inmates by training them to educate and support other inmates is an important step in managing and curbing HIV in prison environments (34).

WLWH also described the difficulty of disclosing their status to their family and friends while living in their home environment because they felt isolated and afraid of being labelled, judged or rejected. They stated that although HIV was prevalent in their communities, most people did not openly discuss the illness. Many lacked knowledge about HIV, particularly with regards to transmission. The study from Lesotho supported this finding and also described the dominant misperceptions and ignorance about HIV transmission prevalent in the community, which was another factor responsible for driving the HIV pandemic in Lesotho (29). Lack of knowledge and misperceptions about HIV inevitably led to stigma and discrimination, both verbal and non-verbal, which the WLWH in our study encountered regularly. Understanding HIV stigma is crucial to understanding HIV disclosure. The study in the North West province of SA, which was conducted in both rural and urban settings, demonstrated the high prevalence of HIV stigma that still exists and its inter-relationship with disclosure (31). The fear of stigma, discrimination, rejection and loss of relationships was cited in our study as reasons for WLWH not being able to disclose their status while living in their home environment. This was consistent with a systematic review of community studies from Nigeria (35). Stigma has been associated with negative consequences which include poor treatment adherence and adverse mental health effects (36, 37). This was also found in our study where WLWH felt the need to conceal their status for fear of being stigmatized. This compromised their treatment adherence outside of prison.

A recent social anthropological study in Kerala, India, found that intense, pervasive and multi-faceted stigma against PLWHA still exists in Indian society (38). PLWHA, like persons of lower caste in traditional Brahmanic systems in the region, are subjected to touch aversion, regimes of commensality and marital exclusion. They are also subjected to derogatory labels, being referred to as immoral and impure by HIV-negative individuals. This resonates with the findings of our study where WLWH described similar experiences of enacted stigma.

Some WLWH expressed that although HIV was prevalent in their communities, people avoided discussing it openly. This has also been expressed in other South African studies (39) and underscores the importance of advocating for direct public discourse on HIV/AIDS through education, awareness programmes and support organizations.

Contrary to their home environment, the WLWH described HIV disclosure in prison as being much easier for them. Although many WLWH felt that they were almost forced to disclose due to the lack of privacy in the prison environment, most felt this had a positive effect, as they realised they were not alone. They felt supported by fellow inmates and staff which had a positive impact on them. They described the beneficial effect that disclosure and support had on treatment adherence and health outcomes and discussed the important lifestyle changes they decided to make to live healthier lives. This is in contrast to a recent study conducted in a correctional facility in the United States of America, by Kutnick and colleagues, in which Black and Latin American prisoners spoke about feeling uncomfortable disclosing their HIV status in prison because they felt stigmatised, unsupported and discriminated against (40).

Finally, understanding the cultural context of WLWH is integral to understanding their experiences of contracting and living with HIV. Studies have found that cultural sensitivity is increasingly recognized as a means to enhance the effectiveness of health promotion programmes universally (18). Delivering HIV/AIDS programmes to incarcerated populations should occur within a culturally-informed framework to encourage optimal engagement with inmates. This was evident in our study which highlighted the importance of understanding inmate's cultural beliefs and backgrounds.

## Conclusion

The outcomes of this study revealed that HIV is prevalent in the female inmate population at this correctional centre in KZN, SA and that it has a significant impact on these women's lives biologically, psychologically and interpersonally. The multitude of challenges they face, particularly in their home environment, are highlighted. In contrast, this study underscores the support participants received in the prison setting. Thus, whilst incarcerated, attempts should be made to effectively support and manage the impact of HIV in inmates, which is a view that is supported by international literature (41–43). Further research should aim at exploring such strategies. If female inmates receive HIV education and training, not only can they engage in peer-based HIV education while incarcerated, but they can also form support groups to help other incarcerated WLWH to cope with their illness. More importantly, upon re-entry into their home communities they will be armed with the necessary knowledge and skills to successfully manage their own illness and to impact positively on the lives of other WLWH in their communities. This would play a pivotal role in curbing the epidemic, since the importance of educating society about HIV regarding causality and transmission, in order to eradicate misconceptions, stigma and discrimination as well as to encourage disclosure and health-seeking behaviour, has also been emphasized. Due to differing inmate profiles in other correctional centres in SA, the authors recommend that similar studies be conducted at these various centres in order to compare findings, and to serve as an evidence base for the development of national rehabilitation programmes aimed at addressing these challenges.

## Limitations

The study was conducted at one correctional centre in SA. The home language for the majority of the women in the study was isiZulu, however, all qualitative interviews were conducted in English. Hence, it is possible that subtle nuances in the narratives might have been missed. The first author was also aware of the potential for asymmetry in the power dynamics between the interviewer and the participants, as the interviewer was a psychiatrist. Therefore, confidentiality, anonymity and the fact that the researcher was independent of the DCS was emphasised to participants. In addition, participants were informed that the first author could in no way influence their criminal proceedings. Lastly, there were limited qualitative studies for comparison, on the lived experiences of HIV in female inmates with a lifetime history of mental illness.

## Data Availability Statement

The raw data supporting the conclusions of this article will be made available by the authors, without undue reservation.

## Ethics Statement

The studies involving human participants were reviewed and approved by Human Research Ethics Committee: University of the Witwatersrand. The participants provided their written informed consent to participate in this study.

## Author Contributions

SN conceptualized the study, collected and analysed the data, wrote the manuscript, and obtained funding. LF, US, and SP were involved in conception of the study protocol, supervision, and editing of the manuscript. All authors contributed to the article and approved the submitted version.

## Conflict of Interest

The authors declare that the research was conducted in the absence of any commercial or financial relationships that could be construed as a potential conflict of interest. The reviewer JJD declared a shared affiliation with several of the authors, SN, LF, US, to the handling editor at time of review.

## Publisher's Note

All claims expressed in this article are solely those of the authors and do not necessarily represent those of their affiliated organizations, or those of the publisher, the editors and the reviewers. Any product that may be evaluated in this article, or claim that may be made by its manufacturer, is not guaranteed or endorsed by the publisher.
